# The Role of Dopamine in Schizophrenia from a Neurobiological and Evolutionary Perspective: Old Fashioned, but Still in Vogue

**DOI:** 10.3389/fpsyt.2014.00047

**Published:** 2014-05-19

**Authors:** Ralf Brisch, Arthur Saniotis, Rainer Wolf, Hendrik Bielau, Hans-Gert Bernstein, Johann Steiner, Bernhard Bogerts, Katharina Braun, Zbigniew Jankowski, Jaliya Kumaratilake, Maciej Henneberg, Tomasz Gos

**Affiliations:** ^1^Department of Forensic Medicine, Medical University of Gdańsk, Gdańsk, Poland; ^2^School of Medical Sciences, The University of Adelaide, Adelaide, SA, Australia; ^3^Centre for Evolutionary Medicine, University of Zurich, Zurich, Switzerland; ^4^Department of Psychiatry and Psychotherapy, Ruhr University Bochum, Bochum, Germany; ^5^Department of Psychiatry, Otto-von-Guericke-University of Magdeburg, Magdeburg, Germany; ^6^Department of Zoology, Institute of Biology, Otto-von-Guericke-University of Magdeburg, Magdeburg, Germany; ^7^Biological Anthropology and Comparative Anatomy Research Unit, School of Biomedical Sciences, The University of Adelaide, Adelaide, SA, Australia

**Keywords:** dopamine, schizophrenia, cognition, glutamate, dopamine receptors, cannabis, animal models of schizophrenia, evolution of the human brain

## Abstract

Dopamine is an inhibitory neurotransmitter involved in the pathology of schizophrenia. The revised dopamine hypothesis states that dopamine abnormalities in the mesolimbic and prefrontal brain regions exist in schizophrenia. However, recent research has indicated that glutamate, GABA, acetylcholine, and serotonin alterations are also involved in the pathology of schizophrenia. This review provides an in-depth analysis of dopamine in animal models of schizophrenia and also focuses on dopamine and cognition. Furthermore, this review provides not only an overview of dopamine receptors and the antipsychotic effects of treatments targeting them but also an outline of dopamine and its interaction with other neurochemical models of schizophrenia. The roles of dopamine in the evolution of the human brain and human mental abilities, which are affected in schizophrenia patients, are also discussed.

## Brief History of Dopamine Hypothesis in Schizophrenia

Dopamine, adrenaline, and noradrenaline are neurotransmitters that belong to the catecholamine family. Dopamine is produced in the substantia nigra and ventral tegmental regions of the brain, and dopamine alterations are related to schizophrenia (1, 2). Dopaminergic projections are divided into the nigrostriatal, mesolimbic, and mesocortical systems. Impairments in the dopamine system result from dopamine dysfunctions in the substantia nigra, ventral tegmental region, striatum, prefrontal cortex, and hippocampus (3–5). The “original dopamine hypothesis” states that hyperactive dopamine transmission results in schizophrenic symptoms. This hypothesis was formed upon the discovery of dopamine as a neurotransmitter in the brain by Arvid Carlsson (6–12). Dopamine receptor blockade by chlorpromazine and haloperidol, proposed in 1963 by Arvid Carlsson and Margit Lindqvist, was a cornerstone in psychiatry (13). However, the association between schizophrenic symptoms and dopamine over-activity has already been questioned (14). The positive symptoms of schizophrenia include hallucinations and delusions as a result of increased subcortical release of dopamine, which augments D_2_ receptor activation (15), and are thought to be due to a disturbed cortical pathway through the nucleus accumbens (16). The negative symptoms of schizophrenia include anhedonia, lack of motivation, and poverty of speech, which result from reduced D_1_ receptor activation (15) in the prefrontal cortex and decreased activity of the nucleus caudatus (16). Alterations in D (3)-receptors might also be involved in the negative symptoms of schizophrenia (17). Furthermore, dopaminergic and serotonergic deviations are known to contribute significantly to both the positive and negative symptoms of schizophrenia [review by Davis et al. (18); Castner and Goldman-Rakic (19); Carlsson et al. (20)].

The “revised dopamine hypothesis” proposes hyperactive dopamine transmission in the mesolimbic areas and hypoactive dopamine transmission in the prefrontal cortex in schizophrenia patients (21–23). In addition to the mesolimbic brain areas, dopamine dysregulation is also observed in brain regions including the amygdala and prefrontal cortex, which are important for emotional processing (24). PET-studies (positron emission tomography) have identified differences in dopamine contents in the prefrontal cortex, cingulate cortex, and hippocampus between schizophrenia patients and neuropsychiatric healthy control subjects (25). In particular, the dopamine system in the hippocampus is overactive in schizophrenia patients [review by Grace (26)].

## Recent Animal Models Implicating Dopamine in Schizophrenia

The prepulse inhibition (PPI) of the acoustic SR (ASR) is a neurophysiologic measurement of sensorimotor gating and a marker for information-processing deficits in neuropsychiatric disorders such as schizophrenia (27–30). PPI refers to a reduced startle response to a strong sensory stimulus when the stimulus is preceded by a barely detectable stimulus (i.e., the prepulse). PPI is similar in human and experimental animal models. Deficits in PPI can be produced in rodents by administering psychotomimetics such as dopaminergic and serotonergic agonists and glutamatergic antagonists (31–34). Furthermore, dopaminergic stabilizers have been shown to restore social behavior in a rat model of schizophrenia (35), and regulatory feedback loops exist among serotonergic, GABAergic, and dopaminergic neurotransmitters (36). For example, interactions between accumbal dopamine and various non-dopamine receptors, such as *N*-methyl-d-aspartate (NMDA)-, AMPA-, GABA (A)-, and nicotinic-receptors were reported in a rodent model of schizophrenia (37). NMDA- and D (1)-receptors in the nucleus accumbens interact with other, and this interaction is controlled by PPI (38). GABA is also involved in the pathophysiology of PPI. Pregnenolone, a neurosteroid in the central nervous system (CNS), works by improving cognitive deficits through GABA, and pregnenolone improves PPI deficits in dopamine transporter knockout mice (39). Activation of GABA-receptors in the rat brain results in various receptor interactions with glutamate (40, 41), and modulation of GABA (A) a5 receptors improves cognitive deficits in rats (42). In a rat model of schizophrenia, the increase in dopamine is caused by hyperactivity of the ventral hippocampus (43). Changes in dopamine receptors (D-2) caused by antipsychotic drugs, such as quinpirole, have been demonstrated in a validated rodent model of schizophrenia (44, 45). However, recent work by Bay-Richter et al. (46) indicates that antipsychotic drugs, such as AMP, clozapine, and haloperidol, cause behavioral changes independent of D (2)-receptors in a mouse model. An up-regulation of D2-high receptors is a consistent feature in animal models of schizophrenia (47). However, alterations in D (3) dopamine receptors caused by novel antipsychotic drugs, such as cariprazine, decrease cognitive deficits in knockout mice (48). Therefore, D (3)-receptor antagonists are recommended as a new pharmacological strategy to improve cognitive function in schizophrenia [review by Nakajima et al. (49)]. Social isolation rearing in rats, which is a valid neurodevelopmental model of schizophrenia, reduces dopamine levels in the frontal cortex (50).

## Cognition in Schizophrenia

Cognitive deficits in schizophrenia affect working memory, language and executive function, episodic memory, processing speed, attention inhibition, and sensory processing (51). The prefrontal region is affected in cognitive discrepancies connected with working memory [see the systematic review by Smieskova et al. (52)], which consists of visual, verbal, central executive, episodic components, and working memory disturbances in schizophrenia are primarily due to altered dorsolateral prefrontal cortex (DLPFC) function (51). Episodic memory discrepancies in schizophrenia involve the medial temporal cortex, particularly the hippocampus, and the prefrontal cortex, particularly the ventral and dorsolateral prefrontal regions (51). Additionally, auditory processing involving memory procedures is impaired in the working memory of schizophrenia patients (53). Cognitive deficits correlate with a decline in dopamine in the prefrontal cortex, primarily at the level of D (1)-receptors (54–59) but also due an imbalance of D (1) and D (2)-receptors in the prefrontal cortex [review by Durstewitz and Seamans (60); Takahashi (61)]. Several studies have proposed that an inverted U-shaped relation between working memory and activation of the prefrontal cortex exists in schizophrenia patients (62). There is ongoing discussion regarding the involvement of D (1)- and D (2)-receptors in cognition in schizophrenia patients (63–66). Cognitive discrepancies and working memory deficits in the prefrontal cortex are associated with an increase in dopamine and D (1)-receptors in the prefrontal cortex in schizophrenia patients (67, 68). Atypical antipsychotics such as clozapine block D (2)-receptors in the striatum and 5-HT_1A_-receptors in the prefrontal cortex, which results in increased dopamine activity (69, 70). By blocking D (2)-receptors through antipsychotics, the apoptotic mechanisms in the brain regions involved in cognition are impaired (71). The disturbed activity of working memory in the DLPFC in schizophrenia patients is influenced by the release of dopamine in the midbrain in schizophrenia patients, which is regulated by a deficit in glutamatergic projection from the DLPFC to midbrain dopamine neurons (72). Extrastriatal dopamine transmission is necessary for attention and working memory, and these deficits in the fronto–striato–thalamic pathway are involved in cognition in schizophrenia (73). Newer antipsychotic drugs such as olanzapine and clozapine, which have a better affinity for dopamine receptors and blocking 5-HT_2A_ receptors, decrease the hyperactivity of the mesolimbic dopaminergic pathway and improve the activity of D (1)-receptors in the prefrontal cortex (74). Furthermore, nicotine improves cognition in schizophrenia patients (75).

The COMT-Val-allele leads to a deficit in cognitive abilities. Interactions between dopaminergic and methylation mechanisms may result in cognitive deficits in schizophrenia patients. The COMT Met-allele results in lower COMT-activity, leading to greater production of dopamine and increased D (1)-receptor activity in the prefrontal cortex and, subsequently, better cognitive abilities in carriers of the Met-allele (76–82). A link between Met-carriers and smoking has been recently reviewed (83), and an association between COMT and cognitive dysfunction in bipolar disorder has also been discussed (84). The COMT-alleles are composed of two different alleles that result in varied activity levels: the low-activity COMT-allele (L-COMT) and the high-activity COMT-allele (H-COMT) (85). The L-COMT allele has the Met-/Met-genotype, and the H-COMT allele has the Val-/Val-genotype (86). Middle-aged healthy women with H-COMT who carry the Val158 allele show better cognitive abilities, including executive processing and cognitive flexibility, than carriers of the Met allele (87).

## Dopamine Receptors and Antipsychotic Effects in Schizophrenia

Dopamine receptors are G-protein-coupled receptors and can be divided into D (1), D (2), D (3), and D (4)-receptors (88). D (1) receptors in the prefrontal cortex are decreased in schizophrenia patients and are unaffected by chronic treatment of typical neuroleptics [review by Friedmann et al. (89)]. In contrast, D (1)-receptors are increased in the parieto-temporal cortex in schizophrenia patients (90). Increased D2 mRNA has been found in the frontal cortex in schizophrenia patients when compared with neuropsychiatric healthy control subjects (91). Both the classic and -current antipsychotic drugs act primarily by increasing high-affinity D (2)-receptor expression (92–98). Haloperidol has been shown to increase high-affinity D (2)-receptors in dopamine-sensitive rats in an animal of schizophrenia (99). Dopamine agonists bind to D (2)-high and D (2)-low-receptors (93, 100). This D (2) two-state model is still controversial, although discussions tend to doubt its validity, as demonstrated by *in vitro* binding experiments (101). The action of dopamine agonists is related to dopamine hyperactivity in psychosis (102). Dopamine antagonists and, to a lesser extent, dopamine agonists increase the D (2)-high-receptors (103). This increase in D (2)-high-receptors is a necessary basic requirement for the development of a psychosis that correlates with dopamine supersensitivity (104). This specific increase in D (2)-receptors and dopamine supersensitivity might result in antipsychotic treatment failure (105, 106). Although D (2)-receptor antagonists induce dopamine activity (107), the mechanisms underlying the action of dopamine D (2)-receptor antagonists are not entirely clear. The low therapeutic advantage of dopamine D (2)-receptor antagonists and their high pharmacological selectivity require future research (108). Antipsychotic drugs block D (2) receptors and increase the release of glutamate in the striatum (109), particularly on the right side of the striatum, which is a brain region involved in cognition and reward motivation (110). Glutamate agonists have an effect on D (2) high-receptors in schizophrenia (111, 112). For example, alterations in D (2)-receptor function caused by antipsychotic medication in a rodent model of schizophrenia (44) or by the application of an amphetamine in schizophrenia patients (113) have been recently demonstrated. A D (2)-receptor occupancy of 80% is considered essential for the positive effects of antipsychotic medication (114, 115), whereas continuous high D (2)-receptor occupancy is not required [review by Kapur and Seeman (116); Remington and Kapur (117), systematic review by Uchida et al. (118); Seeman (119)]. The atypical antipsychotic clozapine results in a lower D (2) receptor occupancy than 80% but still has positive effects [review by Nord and Farde (120)]. Schizophrenia patients with extrapyramidal syndromes (EPSs) show an increased D (2)-receptor occupancy (above 80%) in comparison with schizophrenia patients with a good clinical response and no EPSs (i.e., receptor occupancy of 65–80%) [review by Nord and Farde (120)]. Lower doses of antipsychotics such as risperidone are effective and do not induce EPSs (121, 122). This specific D (2)-receptor occupancy in the striatum in schizophrenia patients interacts with the antagonistic effects of 5-HT_2A_ receptors [review by Pani et al. (123)]. D (1)-receptors and NMDA-receptors cooperate with each other (124). Furthermore, the intensification of D (2)-receptor antagonists by D (1)-receptor agonists results in better NMDA transmission, exemplified by the action of clozapine as a partial D (1)-receptor agonist (109). NMDA and D (1) dopamine receptor interaction occurs through signal transduction and phosphorylation and dephosphorylation mechanisms (125). D (1)-receptors are present in GABAergic interneurons (54). For example, valproic acid affects GABA and, subsequently, dopamine (126).

A slightly increased density of D (2)-receptors in basal condition and a significant increase in D (2)-receptors in the striatum of schizophrenia patients has been found (127). This increase of striatal dopamine D (2)-receptors in schizophrenia has also been demonstrated in neuroimaging and molecular imaging studies (128, 129). Specific neurotransmitter pathways such as those of glutamate, GABA, and acetylcholine lead to a high-affinity of the D (2)-receptor (130). Dopamine receptors such as the D (2)-receptor contain receptor mosaics (i.e., RM; dimeric or high-order receptor oligomers). These D_2_/NMDA receptor mosaics have also been found in the ventral striato-pallidal GABA neurons. Decreased D (2)-receptors in the thalamus and anterior cingulate cortex in schizophrenia might suggest that they are involved in abnormalities in dopamine transmission from the thalamus to the prefrontal cortex (131).

Low doses of D (2)-receptor antagonists and signaling enhancers of NMDA-receptors are recommended as new treatments in schizophrenia [review by Fuxe et al. (132)]. In the associative striatum, an increased D (2)-receptor availability has been found in schizophrenia patients (127). Increased dopamine release in the striatum is linked to substance dependence, such as amphetamine dependency, in schizophrenia (133). For example, stimulation of NMDA/AMPA and kainate receptors by direct application of glutamate or glutamate agonists increases the dopaminergic cell-firing rate (133). However, the role of dopamine in the dysfunction of the striatum in schizophrenia patients requires future research (134).

It can be summarized that, to date, the mechanism of every effective antipsychotic medication in schizophrenia involves dopamine and its interaction with other neurochemical pathways such as those of glutamate, GABA, serotonin, and acetylcholine.

## Alternate Neurochemical Models in Schizophrenia and Their Interactions with Dopamine

Deviations in dopamine and glutamate have been reported in the prefrontal cortex of schizophrenia patients (135). NMDA-receptors are involved in releasing dopamine into the striatum and frontal cortex in schizophrenia patients [Ref. (136, 137), review by Castner and Williams (138); Javitt (139); Balla et al. (140); Laruelle (141)] and in rats in an animal model of schizophrenia (142). These interactions are accompanied by calcium-dependent changes (143) and exchanges between DAT and G72 in various brain regions (144). In contrast to dopamine receptors, glutamate receptors are found in the subcortical and cortical brain regions (145). The activity of dopamine is regulated by GABA and glutamate. For example, corticostriatal glutamatergic pathways interact with dopamine terminals (146, 147), and specific glutamate receptors in the striatum, such as mGlu2, are sensitive to dopamine (112). High glutamate levels have been found in the dorsal caudate nucleus of schizophrenia patients (148). Adenosine interacts with glutamate, NMDA-receptors, and dopamine [review by Burnstock et al. (149)]. It can be summarized that NMDA-receptors and D (1)-receptors in cortical brain areas such as the prefrontal cortex and an excess of D (2)-receptors in subcortical brain areas such as the striatum are interconnected with each other through a positive feedback mechanism (150). However, through its presynaptic action, dopamine reduces the release of glutamate in the pyramidal neurons of layer V in the prefrontal cortex (151). Dopamine dysregulation in the basal ganglia of schizophrenia patients is an important intrinsic feature in the pathology of schizophrenia and not a medication side effect [review by Perez-Costas et al. (3)].

The finding by Brisch et al. (152) that astrocyte density is increased in the frontal cortex in schizophrenia suggests a disturbance in the dopamine–glutamate function. Furthermore, Sokoloff et al. (153) demonstrated that D (3)-receptors either act directly on NMDA-receptors at glutamate synapses on the terminals of pyramidal cells in the nucleus accumbens or act indirectly through dopamine at the presynaptic junction to regulate pyramidal cells in the prefrontal cortex. Indeed, injection of NMDA-antagonists such as MK801 increases glutamate concentration in the frontal, retrosplenial, and cingulate cortices (154). Glutamate dysfunction in the prefrontal cortex and hippocampus causes the release of dopamine in the striatum (155). A new focus on glutamatergic signaling mediated by NMDA and metabotropic receptors may benefit new drug developments [review by Field et al. (156); Javitt (139); Matosin and Newell (157); Moghadam and Krystal (158); Noetzel et al. (159)]. The review by Bernstein et al. (160) attributes the disturbed function of astrocytes in schizophrenia to diminished glutamate metabolism. The enzyme glutamine synthetase, which degrades glutamate into glutamine, is located in glial cells and is decreased in schizophrenia patients (161). Additionally, the glutamate transporter for astrocytes, GLT-1, is increased in schizophrenia patients (162). Although Arai et al. (163) reported no association between glutamine synthetase and schizophrenia, the enzyme glutamine synthetase displays gender-specific differences in schizophrenia (164) and is involved in suicidal behavior (165, 166). Moreover, the atypical antipsychotic agent risperidone increases glutamine synthetase levels (167).

Through NMDA-stimulated GABA-release and GABA_B_-receptor activity, glycine reduces the release of dopamine by modulating DAT-type transporters in the prefrontal cortex and striatum (168). The GABA_B_-receptor inhibits the release of glutamate in the ventral tegmental area (169). A synergistic interaction of adenosine and glutamate affecting the ventral striato-pallidal GABA pathway has been demonstrated in a rat model (170). The interactions of pyramidal neurons with dopamine receptors on their dendrites and pyramidal cells with glutamate on their spines, and GABAergic interneurons in the prefrontal cortex in schizophrenia patients might offer new insights into receptor-targeted therapies [Ref. (53); review by Wassef et al. (171); Lisman et al. (172)]. An increased number of GABA-cells expressing D (1)-receptors exists in the rat prefrontal cortex (173). In the nucleus accumbens, neurotensin (NT) inhibits dopamine discharge, which increases glutamate release and activates the ventral striato-pallidal GABA pathway, leading to a subsequent increase in glutamate transport from the mediodorsal thalamus to the prefrontal cortex (174). Another interaction between dopamine in the prefrontal cortex and glutamate in the mediodorsal thalamus might be responsible for the effects of zotepine, which increases the extracellular levels of noradrenaline, dopamine, glutamate, and GABA (175). GABA interacts with acetylcholine by constraining its excitatory contribution to cholinergic interneurons, which are decreased in the striatum of schizophrenia patients, resulting in prefrontal deviations in schizophrenia (176). Dopamine also interacts with acetylcholine, which increases with smoking frequency in schizophrenia patients (177). Acute nicotine administration might have positive effects on cognition in schizophrenia patients [Ref. (178, 179), review by Mackowick et al. (180)].

Dopamine neurons in the midbrain release serotonin, which is important during combined drug treatment with serotonin to prevent the so-called serotonin syndrome, a surplus of serotonin in some brain regions (181). Atypical antipsychotics involving serotonin receptors include 5-HT_1A_ receptor agonists or antagonists, 5-HT_2A_ receptor antagonists, 5-HT_2c_ receptor inverse or partial agonists or neutral antagonists, 5-HT_6_ receptor antagonists, and 5-HT_7_ receptor antagonists (182).

Antipsychotics (such as clozapine and aripiprazole) possessing 5-HT-_1A_ agonist properties induce hippocampal neurogenesis and increase dopamine in the prefrontal cortex [review by Schreiber and Newman-Tancredi (183)]. It can be summarized that various serotonin–dopamine interactions, which include both direct and indirect feedback mechanisms, contribute to the pathology of schizophrenia [Ref. (151, 184–189), review by Arranz and de Leon (190); Alex and Pehek (191); McCreary et al. (192); Bhattacharyya et al. (193); Meltzer et al. (194); McMahon and Cunningham (195); Gao et al. (196)].

Novel antipsychotic drugs, such as asenapine, increase dopamine and glutamate levels in various subcortical and cortical areas (197). New antipsychotic drugs with novel mechanisms induce alterations in both dopamine and glutamate [review by Paz et al. (198); Seeman et al. (99); Stone (199); Leroy et al. (200); Coyle et al. (201)]. For example, metabotropic glutamate and NMDA-receptors are future targets for new drugs (202, 203). A range of dopamine/serotonin, glutamate/serotonin, and acetylcholine/serotonin interactions activate receptors and signaling molecules in response to antipsychotic drugs and have been observed in various brain regions, including the prefrontal cortex and limbic regions, in schizophrenia (20, 98, 176, 182, 194, 204–211). Future drug development should target signaling molecules involved in dopamine, glutamate, and serotonin neurotransmission such as Akt and glycogen synthase kinase-3 (98, 212, 213) as well as the control of presynaptic dopamine synthesis and release (114). Stress in schizophrenia patients causes an increased release of dopamine in the prefrontal cortex, which cannot be counteracted by reduced GABA_A_ receptor complex activity, as well as dendritic spine loss in the prefrontal cortex (214, 215). When used in schizophrenic patients, cannabis induces hyperdopaminergic and hypoglutamatergic activities with both positive and negative symptoms (216). In particular, cannabis increases dopamine transmission in the nucleus accumbens, which might cause or aggravate psychoses (217). A high-low activity polymorphism in COMT interacts with adolescent cannabis abuse, increasing the risk for schizophrenia (218). Further, genes such as disrupted-in-schizophrenia-1 (DISC1) play a role in stress pathways and the metabolism of dopamine in schizophrenia [review by Hains and Arnsten (219); Lipina et al. (220)].

## Dopamine and Human Evolution

The role of dopamine in human evolution has hitherto received little theoretical attention. It is still unclear to what extent dopaminergic expansion in hominin evolution was due to genetic adaptations or epigenetic factors. Dopamine has expanded throughout primate and hominin evolution and that dopamine is especially concentrated in the prefrontal cortex, which is involved in higher order functioning. The dopaminergic hypothesis contends that climatic changes occurring in sub-Saharan Africa during the Pliocene and Pleistocene periods, which resulted in increases of the Savannah belt expanded hominin locomotory range. It is also speculated that some human groups ventured to the more habitable African southern coast leading to dietary changes (i.e., increasing amounts of fish/shellfish) that aided dopaminergic expansion (221–223). Dopamine increase may have also been linked with a concomitant elevation in thyroid hormone production. Higher T4 found in *Homo* may have represented an early endocrinological difference between humans and other primates (224). In humans, T4 concentration is associated with tyrosine conversion to dopa (a precursor to dopamine); deficiencies of T4 concentrations are linked with various neurological impairments (224).

Recent research suggests that from *Homo erectus* onward, humans became persistence hunters, requiring various morphological and thermo-regulatory modifications (i.e., vascular reactivity to temperature, large body surface area, plantar arch), which provides approximately 20% energy return during running, elastic tendons, short toes, more pronounced *gluteus maximus* muscle, long legs, CNS coordination of metabolic, and cardio-vascular responses to sustained running (225). From *Homo erectus* onward there is an evident increase in stride size, which also optimized ergonomic requirements of bipedalism while diminishing energy requirements. Greater mass of slow twitch muscles would have also assisted long distance locomotion. Long distance locomotion in conjunction with greater hunting activities in ancestral hominins incorporated all aspects of the CNS such as retention and memory recall of large geographic areas, which maximized resource acquisition. The locomotion/behavior interplay, which was mediated by nerve cells and the dopaminergic system may have evolutionary expanded cortical regions and neuro-hormonal organization in ancestral hominins (225).

## Conflict of Interest Statement

The authors declare that the research was conducted in the absence of any commercial or financial relationships that could be construed as a potential conflict of interest.

## Abbreviations

AMPA, (S)-2-amino-3-(3-hydroxy-5-methyl-4-isoxazolyl)-propionic acid; COMT, catechol-*O*-methyltransferase; CNS, central nervous system; D (1), dopamine D_1_ receptor; D (2), dopamine D_2_ receptor; D (3), dopamine D_3_ receptor; DAT, dopamine transporter; DAOA or G72, d-amino acid oxidase activator; DLPFC, dorsolateral prefrontal cortex; GABA, γ-aminobutyric acid; H-COMT, high-activity catechol-*O*-methyltransferase; L-COMT, low-activity catechol-*O*-methyltransferase; Met, methionine; mRNA, messenger ribonucleic acid; NMDA-receptor, *N*-methyl-d-aspartate receptor; PET, positron emission tomography; PPI, prepulse inhibition; RNA, ribonucleic acid; SR, startle reflex; Val, valine.

## References

[1] Bogerts B Häntsch J Herzer M A morphometric study of the dopamine-containing cell groups in the mesencephalon of normals, Parkinson patients, and schizophrenics. Biol Psychiatry (1983) 18:951–69 6640008

[2] Wang YC Ho UC Ko MC Liao CC Lee LJ Differential neuronal changes in medial prefrontal cortex, basolateral amygdale and nucleus accumbens after postweaning social isolation. Brain Struct Funct (2012) 217:337–51 10.1007/s00429-011-0355-4 22002740

[3] Perez-Costas E Melendez-Ferro M Roberts RC Basal ganglia pathology in schizophrenia: dopamine connections and anomalies. J Neurochem (2010) 113:287–302 10.1111/j.1471-4159.2010.06604.x 20089137 PMC2929831

[4] Lodge DJ Grace AA Hippocampal dysregulation of dopamine system function and the pathology of schizophrenia. Trends Pharmacol Sci (2011) 32:507–13 10.1016/j.tips.2011.05.001 21700346 PMC3159688

[5] Yoon JH Minzenberg MJ Raouf S D’Esposito M Carter CS Impaired prefrontal-basal ganglia functional connectivity and substantia nigra hyperactivity in schizophrenia. Biol Psychiatry (2013) 74(2):122–9 10.1016/j.biopsych.2012.11.018 23290498 PMC3620727

[6] Carlsson A Lindqvist M Effect of chlorpromazine or haloperidol on formation of 3methoxytyramine and normetanephrine in mouse brain. Acta Pharmacol Toxicol (Copenh) (1963) 20:140–4 10.1111/j.1600-0773.1963.tb01730.x 14060771

[7] Horn AS Snyder SH Chlorpromazine and dopamine: conformational similarities that correlate with the antischizophrenic activity of phenothiazine drugs. Proc Natl Acad Sci U S A (1971) 68:2325–8 10.1073/pnas.68.10.2325 5289865 PMC389413

[8] Hökfelt T Ljungdahl A Fuxe K Johansson O Dopamine nerve terminals in the rat limbic cortex: aspects of the dopamine hypothesis of schizophrenia. Science (1974) 184:177–9 4856104 10.1126/science.184.4133.177

[9] Iversen SD Iversen LL Dopamine: 50 years in perspective. Trends Neurosci (2007) 30:188–93 10.1016/j.tins.2007.03.002 17368565

[10] Andersen JK Arvid Carlsson: an early pioneer in translational medicine. Sci Transl Med (2009) 1:2s3 10.1126/scitranslmed.3000149 20368162

[11] Yeragani VK Tancer M Chokka P Baker GB Arvid Carlsson, and the story of dopamine. Indian J Psychiatry (2010) 52:87–8 10.4103/0019-5545.58907 20174530 PMC2824994

[12] Madras BK History of the discovery of the antipsychotic dopamine d2 receptor: a basis for the dopamine hypothesis in schizophrenia. J Hist Neurosci (2013) 22:62–78 10.1080/0964704X.2012.678199 23323533

[13] Baumeister AA The chlorpromazine enigma. J Hist Neurosci (2013) 22:14–29 10.1080/0964704X.2012.664087 23323529

[14] Moncrieff J A critique of the dopamine hypothesis of schizophrenia and psychosis. Harv Rev Psychiatry (2009) 17:214–5 10.1080/10673220902979896 19499420

[15] Shen L-H Liao M-H Tseng YC Recent advances in imaging of dopaminergic neurons for evaluation of neuropsychiatric disorders. J Biomed Biotechnol (2012) 2012:1–14 10.1155/2012/259349 22570524 PMC3335602

[16] O’Donnell P Grace AA Dysfunctions in multiple interrelated systems as the neurobiological bases of schizophrenic clusters. Schizophr Bull (1998) 24:267–83 9613625 10.1093/oxfordjournals.schbul.a033325

[17] Simpson EH Winiger V Biezonski DK Haq I Kandel ER Kellendonk C Selective overexpression of dopamine D3 receptors in the striatum disrupts motivation but not cognition. Biol Psychiatry (2013). 10.1016/j.biopsych.2013.11.023 24387821 PMC4047204

[18] Davis KL Kahn RS Ko G Davidson M Dopamine in schizophrenia: a review and reconceptualization. Am J Psychiatry (1991) 148:1474–86 1681750 10.1176/ajp.148.11.1474

[19] Castner SA Goldman-Rakic S Long-lasting psychotomimetic consequences of repeated low-dose amphetamine exposure in rhesus monkeys. Neuropsychopharmacology (1998) 20:10–28 10.1016/S0893-133X(98)00050-5 9885781

[20] Carlsson A Waters N Carlsson ML Neurotransmitter interactions in schizophrenia-therapeutic implications. Biol Psychiatry (1999) 46:1388–95 10.1016/S0006-3223(99)00117-1 10578453

[21] da Silva Alves F Figee M van Amelsvoort T Veltman D de Haan L The revised dopamine hypothesis of schizophrenia: evidence from pharmacological MRI studies with atypical antipsychotic medication. Psychopharmacol Bull (2008) 41:121–32 18362875

[22] Walter H Kammerer H Frasch K Spitzer M Abler B Altered reward functions in patients on atypical antipsychotic medication in line with the revised dopamine hypothesis of schizophrenia. Psychopharmacology (Berl) (2009) 206:121–32 10.1007/s00213-009-1586-4 19521678

[23] Pogarell O Koch W Karch S Dehning S Müller N Tatsch K Dopaminergic neurotransmission in patients with schizophrenia in relation to positive and negative symptoms. Pharmacopsychiatry (2012) 45 (Suppl 1):S36–41 22565233 10.1055/s-0032-1306313

[24] Laviolette SR Dopamine modulation of emotional processing in cortical and subcortical neural circuits: evidence for a final common pathway in schizophrenia? Schizophr Bull (2007) 33:971–84 10.1093/schbul/sbm048 17519393 PMC2632330

[25] Patel NH Vyas NS Puri BK Nijran KS Al-Nahas A Positron emission tomography in schizophrenia: a new perspective. J Nucl Med (2010) 51:511–20 10.2967/jnumed.109.066076 20237027

[26] Grace A Dopamine system dysregulation by the hippocampus: implications for the pathophysiology and treatment of schizophrenia. Neuropharmacology (2012) 62:1342–8 10.1016/j.neuropharm.2011.05.011 21621548 PMC3179528

[27] Geyer MA Braff DL Startle habituation and sensorimotor gating in schizophrenia and related animal models. Schizophr Bull (1987) 13:643–68 10.1093/schbul/13.4.643 3438708

[28] Braff DL Geyer MA Sensorimotor gating and schizophrenia. Human and animal model studies. Arch Gen Psychiatry (1990) 47:181–8 10.1001/archpsyc.1990.01810140081011 2405807

[29] Swerdlow NR Braff DL Geyer MA Cross-species studies of sensorimotor gating of the startle reflex. Ann N Y Acad Sci (1999) 877:202–16 10.1111/j.1749-6632.1999.tb09269.x 10415651

[30] Meincke U Mörth D Voss D Thelen B Geyer MA Gouzoulis-Mayfrank E Prepulse inhibition of the acoustically evoked startle reflex in patients with an acute schizophrenic psychosis-A longitudinal study. Eur Arch Psychiatry Clin Neurosci (2004) 254:415–21 10.1007/s00406-004-0523-0 15538598

[31] Braff DL Geyer MA Swerdlow NR Human studies of prepulse inhibition of startle: normal subjects, patient groups, and pharmacological studies. Psychopharmacology (Berl) (2001) 156:234–58 10.1007/s002130100810 11549226

[32] Geyer MA Krebs-Thomson K Braff DL Swerdlow NR Pharmacological studies of prepulse inhibition models of sensorimotor gating deficits in schizophrenia: a decade of review. Psychopharmacology (Berl) (2001) 156:117–54 10.1007/s002130100811 11549216

[33] Bast T Feldon J Hippocampal modulation of sensorimotor processes. Prog Neurobiol (2003) 70:319–45 10.1016/S0301-0082(03)00112-6 12963091

[34] Kwek P van den Busse M Modafinil disrupts prepulse inhibition in mice: strain differences and involvement of dopaminergic and serotonergic activation. Eur J Pharmacol (2012) 699:132–40 10.1016/j.ejphar.2012.11.041 23219987

[35] Rung JP Carlsson A Markinhuta KR Carlsson ML The dopaminergic stabilizers (-)-OSU6162 and ACR162 reverse (+)-MK-801-induced social withdrawal in rats. Prog Neuropsychopharmacol Biol Psychiatry (2005) 29:833–9 10.1016/j.pnpbp.2005.03.003 15913873

[36] Peselmann N Schmitt A Gebicke-Haerter PJ Zink M Ariprazole differentially regulates the expression of Gad67 and y-aminobutyric acid transporters in rat brain. Eur Arch Psychiatry Clin Neurosci (2013) 263:285–97 10.1007/S00406-012-0367 22968646

[37] Ikeda H Kamei J Koshikawa N Cools AR Nucleus accumbens and dopamine-mediated turning behavior of the rat: role of accumbal non-dopaminergic receptors. J Pharmacol Sci (2012) 120:152–64 10.1254/jphs.12R02CR 23059952

[38] Glass MJ Robinson DC Waters E Pickel VM Deletion of NMDA-NR1 receptor subunit gene in the mouse nucleus accumbens attenuates apomorphine-induced dopamine D1 receptor trafficking and acoustic startle behavior. Synapse (2013) 67:265–79 10.1002/syn.21637 23345061 PMC3894854

[39] Wong P Chang CCR Marx CE Caron MG Wetsel WC Zhang X Pregnenolone rescues schizophrenia-like behavior in dopamine transporter knockout mice. PLoS One (2012) 7:e51455 10.137/journal.pone.0051455 23240026 PMC3519851

[40] Hickey AJ Reynolds JN Beninger RJ Post-weaning social isolation and subchronic NMDA glutamate receptor blockade: effects on locomotor activity and GABA signaling in the rat suggest independent mechanisms. Pharmacol Biochem Behav (2012) 101:231–8 10.1016/j.pbb.2012.01.015 22293110

[41] Roenker NL Gudelsky GA Ahlbrand R Horn PS Richtand NM Evidence for involvement of nitric oxide and GABA(B) receptors in MK-801-stimulated release of glutamate in rat prefrontal cortex. Neuropharmacology (2012) 63:575–81 10.1016/j.neuropharm.2012.04.032 22579658 PMC3392446

[42] Redrobe JP Elster L Frederiksen K Bundgaard C de Jong IE Smith GP Negative modulation of GABAA a5 receptors by RO4938581 attenuates discrete sub-chronic and early postnatal phencyclidine (PCP)-induced cognitive deficits in rats. Psychopharmacology (Berl) (2012) 221:451–68 10.1007/s00213-011-2593-9 22124672

[43] Lodge DJ Grace AA Aberrant hippocampal activity underlies the dopamine dysregulation in an animal model of schizophrenia. J Neurosci (2007) 27:11424–30 10.1523/JNEUROSCI.2847-07.2007 17942737 PMC6673030

[44] Perez SM Lodge DJ Aberrant dopamine d2-like receptor function in a rodent model of schizophrenia. J Pharmacol Exp Ther (2012) 343:288–95 10.1124/jpet.112.193201 22859862 PMC3477214

[45] Simpson EH Waltz JA Kellendonk C Balsam PD Schizophrenia in translation: dissecting motivation in schizophrenia and rodents. Schizophr Bull (2012) 38:1111–7 10.1093/schbul/sbs114 23015686 PMC3494038

[46] Bay-Richter C O’Callaghan MJ Mathur N O’Tuathaigh CM Heery DM Fone KC D-amphetamine and antipsychotic drug effects on latent inhibition in mice lacking dopamine D2 receptors. Neuropsychopharmacology (2013) 38:1512–20 10.1038/npp.2013.50 23422792 PMC3682146

[47] Novak G Seeman P Hyperactive mice show elevated D2high receptors, a model for schizophrenia: calcium/calmodulin-dependent kinase II alpha knockouts. Synapse (2010) 64:794–800 10.1002/syn.20786 20336626

[48] Zimnisky R Chang G Gyertyan I Kiss B Adham N Schmauss C Cariprazine, a dopamine D(3)-receptor-preferring partial agonist, blocks phencyclidine-induced impairments of working memory, attention, set-shifting, and recognition memory in the mouse. Psychopharmacology (Berl) (2013) 226:91–100 10.1007/S00213-012-2896-5 23079899 PMC3572273

[49] Nakajima S Gerretsen P Takeuchi H Carravaggio P Chow T Le Foll B The potential role of dopamine D_3_ receptor neurotransmission in cognition. Eur Neuropsychopharmacol (2013) 23:799–813 10.1016/j.euroneuro.2013.05.006 23791072 PMC3748034

[50] Möller M Du Preez JL Viljoen F Berk M Emsley R Harvey BH Social isolation induces immuniological, and behavioural deficits in rats, and is reversed by clozapine or N-acetyl cysteine. Brain Behav Immun (2012) 30:156–67 10.1016/j.bbi.2012.12.011 23270677

[51] Barch DM Ceasar A Cognition in schizophrenia: core psychological and neural mechanisms. Trends Cogn Sci (2012) 16:27–34 10.1016/j.tics.2011.11.015 22169777 PMC3860986

[52] Smieskova R Marmy J Schmidt A Bendfeldt K Riecher-Rössler A Walter M Do subjects at clinical high risk for psychosis differ from those with a genetic high risk? – A systematic review by structural and functional brain abnormalities. Curr Med Chem (2013) 20:467–81 10.2174/0929867311320030018 23157639 PMC3580804

[53] Goldman-Rakic PS Selemen LD Functional and anatomical aspects of prefrontal pathology in schizophrenia. Schizophr Bull (1997) 23:437–58 10.1093/schbul/23.3.437 9327508

[54] Goldman-Rakic PS Mully III Williams GV D_1_ receptors in prefrontal cells and circuits. Brain Res Rev (2000) 31:295–301 10.1016/S0165-0173(99)00045-4 10719156

[55] Laruelle M Kegeles LS Abi-Dargham A Glutamate, dopamine, and schizophrenia from pathophysiology to treatment. Ann N Y Acad Sci (2003) 1003:138–58 10.1196/annals.1300.063 14684442

[56] Goldman-Rakic PS Castner SA Svensson TH Siever LJ Williams GV Targeting the dopamine D_1_ receptor in schizophrenia: insights for cognitive dysfunction. Psychopharmacology (2004) 174:3–16 10.1007/s00213-004-1793-y 15118803

[57] Castner SA Vosler PS Goldman-Rakic S Amphetamine sensitization impairs cognition and reduces dopamine turnover in primate prefrontal cortex. Biol Psychiatry (2005) 57:743–51 10.1016/j.biopsych.2004.12.019 15820231

[58] Wiliams GV Castner SA Under the curve: critical issues for elucidating D1 receptor function in working memory. Neuroscience (2006) 139:263–76 10.1016/j.neuroscience.2005.09.028 16310964

[59] Arnsten AFT The neurobiology of thought: the groundbreaking discoveries of Patricia Goldman-Rakic 1937-2003. Cereb Cortex (2013) 23:2269–81 10.1093/cercor/bht195 23926115 PMC3767966

[60] Durstewitz D Seamans JK The dual-state theory of prefrontal cortex dopamine function with relevance to catechol-O-methyltransferase genotypes and schizophrenia. Biol Psychiatry (2008) 64:739–49 10.1016/j.biopsych.2008.05.015 18620336

[61] Takahashi H PET neuroimaging of extrastriatal dopamine receptors and prefrontal cortex functions. J Physiol Paris (2013) 107:503–9 10.1016/j.jphysparis.2013.07.001 23851135

[62] Goto Y Otani S Grace AA The Yin and Yang of dopamine release: a new perspective. Neuropharmacology (2007) 53:583–7 10.1016/j.neuropharm.2007.07.007 17709119 PMC2078202

[63] Gross G Drescher KU The role of dopamine d(3) receptors in antipsychotic activity and cognitive functions. Handb Exp Pharmacol (2012) 213:167–210 10.1007/978-3-642-25758-2_7 23027416

[64] Suridjan I Rusjan P Addington J Wilson AA Houle S Mizrahi R Dopamine D2 and D3 binding in people at clinical high risk for schizophrenia. J Psychiatry Neurosci (2012) 37:110181 10.1503/jpn.110181 PMC3581597 23010256

[65] Gross G Wicke K Drescher KU Dopamine D(3) receptor antagonism-still a therapeutic option for the treatment of schizophrenia. Naunyn Schmiedebergs Arch Pharmacol (2012) 386(2):155–66 10.1007/s00210-012-0806-3 23128852

[66] Micheli F Heidbreder C Dopamine D3 receptor antagonists: a patent review (2007-2011). Expert Opin Ther Pat (2013) 23(3):363–81 10.1517/13543776.2013.757593 23282131

[67] Abi-Dargham A Mawlawi O Lombardo I Gil R Martinez D Huang Y Prefrontal dopamine D1 receptors and working memory in schizophrenia. J Neurosci (2002) 122:3708–19 11978847 10.1523/JNEUROSCI.22-09-03708.2002 PMC6758376

[68] Straffella AP Paus T Fraraccio M Dagher A Striatal dopamine release induced by repetitive transcranial magnetic stimulation of the human motor cortex. Brain (2003) 126:2609–15 10.1093/brain/awg268 12937078

[69] Sumiyoshi T Higuchi Y Uehara T Neural basis for the ability of atypical antipsychotic drugs to improve cognition in schizophrenia. Front Behav Neurosci (2013) 7:140 10.3389/fnbeh.2013.00140 24137114 PMC3797421

[70] Artigas F The prefrontal cortex: a target for antipsychotic drugs. Acta Psychiatr Scand (2010) 121:11–21 10.1111/j.1600-0447.2009.01455.x 20059453

[71] Marinescu I Udristoiu I Marinescu D Choroid plexus calcification: clinical, neuroimaging and histopathological correlations in schizophrenia. Rom J Morphol Embryol (2013) 54:365–9 23771083

[72] Tanaka S Dopaminergic control of working memory and its relevance to schizophrenia: a circuit dynamics perspective. Neuroscience (2006) 139:153–71 10.1016/j.neuroscience.2005.08.070 16324800

[73] Cropley VL Fujita M Innis RB Nathan PJ Molecular imaging of the dopaminergic system and its association with human cognitive function. Biol Psychiatry (2006) 59:898–907 10.1016/j.biopsych.2006.03.004 16682268

[74] Peuskens J Demily C Thibaut F Treatment of cognitive dysfunction in schizophrenia. Clin Ther (2005) 27(Suppl A):S25–37 10.1016/j.clinthera.2005.07.015 16198199

[75] Goff DC Hill M Barch D The treatment of cognitive impairment in schizophrenia. Pharmacol Biochem Behav (2011) 99:245–53 10.1016/j.pbb.2010.11.009 21115035 PMC3114283

[76] Bilder RM Volavka J Lachman HM Grace AM The catechol-O-metyltransferase polymorphism: relations to the tonic phasic dopamine hypothesis and neuropsychiatric phenotypes. Neuropsychopharmacology (2004) 29:1953–61 10.1038/sj.npp.1300542 15305167

[77] Diamond A Briand L Fosella J Gehlbach L Genetic and neurochemical modulation of prefrontal cognitive functions in children. Am J Psychiatry (2004) 161:125–32 10.1176/appi.ajp.161.1.125 14702260

[78] Ceaser A Csernansky JG Barch DM COMT influences on prefrontal and striatal blood oxygenation level-dependent responses during working memory among individuals with schizophrenia, their siblings, and healthy controls. Cogn Neuropsychiatry (2013) 18:257–83 10.1080/13546805.2012.698100 23030509 PMC3874114

[79] Lopez-Garcia P Young Espinoza L Molero Santos P Ortuno-Sanchez-Pedreno F Impact of COMT genotype on cognition in schizophrenia spectrum patients and their relatives. Psychiatry Res (2012) 208:118–24 10.1016/j.psychres.2012.0943 23102922

[80] Julio-Costas A Antunes A Lopes-Silva JB Moreira BC Vianna GS Wood G Count on dopamine: influences of COMT polymorphisms on numerical cognition. Front Psychol (2013) 4:531 10.3389/fpsyg.2013.00531 23966969 PMC3744013

[81] Kontis D Theochari E Fryssira H Kleisas S Sofocleous C Andreopoulou A COMT and polymorphisms interaction on cognition in schizophrenia: an exploratory study. Neurosci Lett (2013) 537:17–22 10.1016/j.neulet.2013.01.012 23353103

[82] Tylec A Kucharska-Pietura K Jeleniewicz W Cybulski M Czernikiewicz A Functional polymorphism of genes inactivating biogenic amines and cognitive deficits in paranoid schizophrenia. Psychiatr Pol (2013) 47:197–211 23888755

[83] de la Salle S Smith D Choueiry J Impey D Philippe T Dort H Effects of COMT genotype on sensory gating and its modulation by nicotine: differences in low and high P50 suppressors. Neuroscience (2013) 241:147–56 10.1016/j.neuroscience.2013.03.029 23535252 PMC4534135

[84] Soeiro-de-Souza MG Machado-Vieira R Soares Bio D Do Prado CM Moreno RA COMT polymorphisms as predictors of cognitive dysfunction during manic and mixed episodes in schizophrenia. Bipolar Disord (2012) 14:554–64 10.1111/j.1399-5618.2012.01030.x 22713126

[85] Lotta T Vidgren J Tilgmann C Ulmannen I Melen K Julkunen I Kinetics of human soluble and membrane-bound catechol-O-methytransferase: a revised mechanism and description of the thermolabile variant of the enzyme. Biochemistry (1995) 34:4202–10 10.1021/bi00013a008 7703232

[86] Mattay VS Goldberg TE Fera F Hariri AR Tessitore A Egan MF Catechol-O-methyl-transferase val158-met genotype and individual variation in the brain response to amphetamine. Proc Natl Acad Sci U S A (2003) 100:6186–91 10.1073/pnas.0931309100 12716966 PMC156347

[87] Solis-Ortiz S Perez-Luque E Morado-Crespo L Gutierrez-Munoz M Executive functions and selective attention are favored in middle-aged healthy women carriers of the Val/Val genotype of the catechol-O-methyltransferase gene: a behavioral genetic study. Behav Brain Funct (2010) 6:67 10.1186/1744-9081-6-67 21029471 PMC2987980

[88] Tritsch NX Sabatini BL Dopaminergic modulation of synaptic transmission in cortex and striatum. Neuron (2012) 76:33–50 10.1016/j.neuron.2012.09.023 23040805 PMC4386589

[89] Friedmann JI Temporini H Davis KL Pharmacologic strategies for augmenting cognitive performance in schizophrenia. Biol Psychiatry (1999) 45:1–16 10.1016/S0006-3223(98)00287-X 9894570

[90] Domyo T Kurumaji A Toru M An increase in [^3^H]SCH23390 binding in the cerebral cortex of postmortem brains of chronic schizophrenics. J Neural Transm (2001) 108:1475–84 10.1007/s007020100021 11810409

[91] Tallerico T Novak G Liu ISC Ulpian C Seeman P Schizophrenia: elevated mRNA for dopamine D2_Longer_ receptors in frontal cortex. Brain Res Mol Brain Res (2001) 87:160–5 10.1016/S0169-328X(00)00293-X 11245917

[92] Farde L Brain imaging of schizophrenia – the dopamine hypothesis. Schizophr Res (1997) 28:157–62 10.1016/S0920-9964(97)00121-7 9468350

[93] Seeman P Dopamine D2 receptors as treatment targets in schizophrenia. Clin Schizophr Relat Psychoses (2010) 4:56–73 10.3371/CSRP.4.1.5 20643630

[94] Seeman P All roads to schizophrenia lead to dopamine supersensitivity and elevated dopamine D2 (high) receptors. CNS Neurosci Ther (2011) 17:118–32 10.1111/j.1755-5949.2010.00162.x 20560996 PMC6493870

[95] Boyd KN Mailman R Dopamine receptor signaling and current and future antipsychotic drugs. Handb Exp Pharmacol (2012) 212:53–86 10.1007/978-3-642-25761-2_3 23129328 PMC4711768

[96] Ito H Takano H Arakawa R Takahashi H Kodaka F Takahata K Effects of dopamine D_2_ receptor partial agonist antipsychotic aripiprazole on dopamine synthesis in human brain measured by PET with-[ß-^11^ C]DOPA. PLoS One (2012) 7:e46488 10.1371/journal.pone.0046488 23029533 PMC3460902

[97] Maheux J St-Hilaire M Voyer D Tirotta E Borrelli E Borelli E Dopamine D2 antagonist-induced striatal Nur77 expression requires activation on mGlu5 receptors by cortical afferents. Front Pharmacol (2012) 3:153 10.3389/fphar.2012.00153 22912617 PMC3418524

[98] de Bartolomeis A Buonaguro EF Iasevoli F Serotonin-glutamate and serotonin-dopamine reciprocal interactions as putative molecular targets for novel antipsychotic treatments: from receptor heterodimers to postsynaptic scaffolding and effector proteins. Psychopharmacology (Berl) (2013) 225:1–19 10.1007/s00213-012-2921-8 23179966

[99] Seeman P Schizophrenia model of elevated D2^High^ receptors: Haloperidol reverses the amphetamine-induced elevation in dopamine D2^High^ receptors. Schizophr Res (2009) 109:191–92 10.1016/j.schres.2008.12.024 19171464

[100] Seeman P Glutamate agonist LY404,039 for treating schizophrenia has affinity for the dopamine D2^High^ receptor. Synapse (2009) 63:935–9 10.1002/syn.20704 19588471

[101] McCormick PN Kapur S Seeman P Wilson AA Dopamine D2 receptor radiotracers [^11^C](+)-PHNO and [^3^H] raclopride are indistinguishably inhibited by D2 agonists and antagonists ex vivo. Nucl Med Biol (2008) 35:11–7 10.1016/j.nucmedbio.2007.08.005 18158938

[102] Seeman P Guan H-C Dopamine partial agonist action of (-)OSU6162 is consistent with dopamine hyperactivity in psychosis. Eur J Pharmacol (2007) 557:151–3 10.1016/j.ejphar.2006.11.016 17157291

[103] Seeman P Dopamine D2^High^ receptors moderately elevated by bifeprunox and aripiprazole. Synapse (2008) 62:902–8 10.1002/syn.20557 18792990

[104] Seeman P Schwarz J Chen J-F Szetchman H Perreault M McKnight GS Psychosis pathways converge via D2^High^ dopamine receptors. Synapse (2006) 60:319–46 10.1002/syn.20303 16786561

[105] Samaha A-N Seeman P Stewart J Rajabi H Kapur S Breakthrough dopamine supersensitivity during ongoing antipsychotic treatment leads to treatment failure over time. J Neurosci (2007) 27:2979–86 10.1523/JNEUROSCI.5416-06.2007 17360921 PMC6672560

[106] Samaha A-N Reckless GE Seeman P Diwan M Nobrega JN Kapur S Less is more: antipsychotic drug effects are greater with transient rather than continuous delivery. Biol Psychiatry (2008) 64:145–52 10.1016/j.biopsych.2008.01.010 18295747

[107] Valenti O Cifelli P Gill KM Grace AA Antipsychotic drugs rapidly induce dopamine neuron depolarization block in a developmental rat model of schizophrenia. J Neurosci (2011) 31:1230–8 10.1523/JNEUROSCI.2808-11.2011 21865475 PMC3167216

[108] Bonci A Hopf FW The dopamine D2 receptor: new surprises from an old friend. Neuron (2005) 47:335–8 10.1016/j.neuron.2005.07.015 16055058

[109] Laruelle M Frankle WG Narendran R Kegeles LS Abi-Dargham A Mechanism of action of antipsychotic drugs: from dopamine D_2_ receptor antagonism to glutamate NMDA facilitation. Clin Ther (2005) 27(Suppl A):S16–24 10.1016/j.clinthera.2005.07.017 16198197

[110] Kim J-H Son Y-D Kim H-K Lee S-Y Cho S-E Kim Y-B Antipsychotic-associated mental side effects and their relationship to dopamine D_2_ receptor occupancy in striatal subdivisions. A high-resolution PET study with [^11^C] raclopride. J Clin Psychopharmacol (2011) 31:507–11 10.1097/JCP.0b013e318222353a 21694619

[111] Seeman P Glutamate agonists for schizophrenia stimulate dopamine D2^High^ receptors. Schizophr Res (2008) 99:373–4 10.1016/j.schres.2007.11.007 18077139

[112] Seeman P Dopamine D2^High^ receptors measured ex vivo are elevated in amphetamine-sensitized animals. Synapse (2009) 63:186–92 10.1002/syn.20595 19086090

[113] Wang M Pei L Fletcher PJ Kapur S Seeman P Liu F Schizophrenia, amphetamine-induced sensitized state and acute amphetamine exposure all show a common alteration: increased dopamine D2 receptor dimerization. Mol Brain (2010) 3:25 10.1186/1756-6606-3-25 20813060 PMC2942879

[114] Howes OD Egerton A Allan V McGuire P Stokes P Kapur S Mechanisms underlying psychosis and antipsychotic treatment response in schizophrenia. Curr Pharm Des (2009) 15:2550–9 10.2174/138161209788957528 19689327 PMC3687204

[115] Yilmaz Z Zai CC Hwang R Mann R Arenovich T Remington G Antipsychotics, dopamine D_2_ receptor occupancy and clinical improvement in schizophrenia: a meta-analysis. Schizophr Res (2012) 140:214–20 10.1016/j.schres.2012.06.027 22795368

[116] Kapur S Seeman P Does fast association from the dopamine D receptor explain the action of atypical antipsychotics?: A new hypothesis. Am J Psychiatry (2001) 158:360–9 10.1176/appi.ajp.158.3.360 11229973

[117] Remington G Kapur S Antipsychotic medication: how much but also how often? Schizophr Bull (2010) 36:900–3 10.1093/schbul/sbq083 20650931 PMC2930338

[118] Uchida H Takeuchi H Graff-Guerrero A Suzuki T Watanabe K Mamo DC Dopamine D_2_ receptor occupancy and clinical effects. J Clin Psychopharmacol (2011) 31:497–502 10.1097/JCP.0b013e3182214aad 21694629

[119] Seeman P Clozapine, a fast-off-D2 antipsychotic. ACS Chem Neurosci (2014) 5:24–9 10.1021/cn400189s 24219174 PMC3894721

[120] Nord M Farde M Antipsychotic occupancy of dopamine receptors in schizophrenia. CNS Neurosci Ther (2011) 17:97–103 10.1111/j.1755-5949.2010.00222.x 21143431 PMC6493889

[121] Bressan RA Erlandsson K Jones HM Mulligan RS Ell P Pilowsky LS Optimizing limbic selective D2/D3 receptor occupancy by risperidone: a [^123^I]-epidepride SPECT study. J Clin Psychopharmacol (2003) 23:5–14 10.1097/00004714-200302000-00002 12544369

[122] Iyo M Tadokoro S Kanahara N Hashimoto T Niitsu T Watanabe H Optimal extent of dopamine D_2_ receptor occupancy by antipsychotics for treatment of dopamine supersensitivity psychosis and late-onset psychosis. J Clin Psychopharmacol (2013) 33:398–404 10.1097/JCP.0b013e31828ea95c 23609386

[123] Pani L Pira L Marchese G Review antipsychotic efficacy: relationship to optimal D_2_-receptor occupancy. Eur Psychiatry (2007) 22:267–75 10.1016/j.eurpsy.2007.02.005 17419008

[124] Kruusmägi M Kumar S Zelenin S Brismar H Aperia A Scott L Functional differences between D 1 and D 5 revealed by high resolution imaging on live neurons. Neuroscience (2009) 164:463–9 10.1016/j.neuroscience.2009.08.052 19723560

[125] Scott L Asperia A Interaction between N-methyl-D-aspartic acid receptors and D1 dopamine receptors: an important mechanism for brain plasticity. Neuroscience (2009) 158:62–6 10.1016/j.neuroscience.2008.10.020 19000746

[126] Lopez LM Wassef AA Molloy MS Williams NG Valproic acid induces manifestations of simultaneous dopamine enhancement and reduction in schizophrenia. Neuropsychopharmacology (2004) 29:127 10.1038/sj.npp.1300461 15154001

[127] Brunelin J Fecteau S Suaud-Chagny MF Abnormal striatal dopamine transmission in schizophrenia. Curr Med Chem (2012) 20:397–404 10.2174/092986713804870819 23157632 PMC3866953

[128] Gur RE Keshavan MS Lawrie SM Deconstructing psychosis with human brain imaging. Schizophr Bull (2007) 33:921–31 10.1093/schbul/sbm045 17548845 PMC2632317

[129] Miyake N Thompson J Skinbjerg M Abi-Dargham A Presynaptic dopamine in schizophrenia. CNS Neurosci Ther (2011) 17:104–9 10.1111/j.1755-5949.2010.00230.x 21199451 PMC6493810

[130] Forti MD Lappin JM Murray RM Risk factors for schizophrenia-all roads lead to dopamine. Eur Neuropsychopharmacol (2007) 17:S101–7 10.1016/j.euroneuro.2007.02.005 17336764

[131] Takahashi H Higuchi M Suhara T The role of extrastriatal dopamine D2 receptors in schizophrenia. Biol Psychiatry (2006) 59:919–28 10.1016/j.biopsych.2006.01.022 16682269

[132] Fuxe K Marcellino D Rivera A Diaz-Cabiale Z Filip M Gago B Receptor-receptor interactions within receptor mosaics. Impact on neuropsychopharmacology. Brain Res Rev (2008) 58:415–52 10.1016/j.brainresrev.2007.11.007 18222544

[133] Kegeles LS Abi-Dargham A Zea-Ponce Y Rodenhiser-Hill J Mann JJ Van Heertum RL Modulation of amphetamine-induced striatal dopamine release by ketamine in humans: implications for schizophrenia. Biol Psychiatry (2000) 48:627–40 10.1016/S0006-3223(00)00976-8 11032974

[134] Heinz A Schlagenhauf F Dopaminergic dysfunction in schizophrenia: salience attribution revisited. Schizophr Bull (2010) 36:472–85 10.1093/schbul/sbq031 20453041 PMC2879696

[135] Rao JS Kellom M Reese EA Rapoport SI Kim HW Dysregulated glutamate and dopamine transporters in postmortem frontal cortex from bipolar and schizophrenic patients. J Affect Disord (2012) 136:63–71 10.1016/j.jad.2011.08.017 21925739 PMC3254216

[136] Kegeles LS Abi-Dargham A Frankle WG Gil R Cooper TB Slifstein M Increased synaptic dopamine function in associative regions of the striatum in schizophrenia. Arch Gen Psychiatry (2010) 67:231–9 10.1001/archgenpsychiatry.2010.10 20194823

[137] Kegeles LS Slifstein M Xu X Urban N Thompson JL Moadel T Striatal and extrastriatal dopamine D2/D3 receptors in schizophrenia evaluated with [18F]fallpride positron emission tomography. Biol Psychiatry (2010) 168:634–41 10.1016/j.biopsych.2010.05.027 20673873 PMC2952433

[138] Castner SA Williams GV Tuning the engine of cognition: a focus on NMDA/D1 receptor interactions in prefrontal cortex. Brain Cogn (2007) 63:94–122 10.1016/j.bandc.2006.11.002 17204357

[139] Javitt DC Glutamatergic theories of schizophrenia. Isr J Psychiatry Relat Sci (2010) 47:4–16 20686195

[140] Balla A Schneider S Sershen H Javitt DC Effects of novel, high affinity glycine transport inhibitors on frontostriatal dopamine release in a rodent model of schizophrenia. Eur Neuropsychopharmacol (2012) 22:902–10 10.1016/j.euroneuro.2012.03.006 22561005 PMC3882073

[141] Laruelle M Schizophrenia: from dopaminergic to glutamatergic interventions. Curr Opin Pharmacol (2014) 14:97–102 10.1016/j.coph.2014.01.001 24524997

[142] Balla A Nattini ME Sershen H Lajtha A Dunlop DS Javitt DC GABA_B_/NMDA receptor interaction in the regulation of extracellular dopamine levels in rodent prefrontal cortex and striatum. Neuropharmacology (2009) 56:915–21 10.1016/j.neuropharm.2009.01.021 19371582 PMC4681299

[143] de Bartolomeis A Tomasetti C Calcium-dependent networks in dopamine-glutamate interaction: the role of postsynaptic scaffolding proteins. Mol Neurobiol (2012) 46:275–96 10.1007/s12035-012-8293-6 22763587

[144] Pauli A Prata DP Mechelli A Picchioni M Fu CH Chaddock CA Interaction between effects of genes coding for dopamine and glutamate transmission on striatal and parahippocampal function. Hum Brain Mapp (2013) 34:2244–58 10.1002/hbm.22061 22438288 PMC6869864

[145] Javitt DC Twenty-five years of glutamate in schizophrenia: are we there yet? Schizophr Bull (2012) 38:911–3 10.1093/schbul/sbs100 22987849 PMC3446216

[146] Juranyi Z Zigmond MJ Harsing LG Jr [^3^H] Dopamine release in striatum in response to cortical stimulation in a corticostriatal slice preparation. J Neurosci Methods (2003) 126:57–67 10.1016/S0165-0270(03)00066-9 12788502

[147] Thompson JL Pogue-Geile MF Grace AA Developmental pathology, dopamine, and stress: a model for the age of onset of schizophrenia symptoms. Schizophr Bull (2004) 30:875–900 10.1093/oxfordjournals.schbul.a007139 15954196

[148] de la Fuente-Sandoval C León-Ortiz P Favila R Stephano S Mamo D Ramírez-Bermúdez J Higher levels of glutamate in the associative-striatum of subjects with prodromal symptoms of schizophrenia and patients with first-episode psychosis. Neuropsychopharmacology (2011) 36:1781–91 10.1038/npp.2011.65 21508933 PMC3154101

[149] Burnstock G Krügel U Abracchio M Illles P Purinergic signalling: from normal behaviour to pathological brain function. Prog Neurobiol (2011) 95:229–74 10.1016/j.pneurobio.2011.08.006 21907261

[150] Abi-Dargham A Laruelle M Mechanisms of action of second generation antipsychotic drugs in schizophrenia. Eur Psychiatry (2005) 20:15–27 10.1016/j.eurpsy.2004.11.003 15642439

[151] Gao W-J Krimer LS Goldman-Rakic PS Presynaptic regulation of recurrent excitation by D1 receptors in prefrontal circuits. Proc Natl Acad Sci U S A (2001) 98:295–300 10.1073/pnas.98.1.295 11134520 PMC14584

[152] Brisch R Bernstein HG Krell D Dobrowolny H Bielau H Steiner J Dopamine-glutamate abnormalities in the frontal cortex associated with the catechol-O-methyltransferase (COMT) in schizophrenia. Brain Res (2009) 1269:166–75 10.1016/j.brainres.2009.02.039 19268435

[153] Sokoloff P Leriche L Diaz J Louvel J Pumain R Direct and indirect pathways of the dopamine D_3_ receptor with glutamate pathways: implications for the treatment of schizophrenia. Naunyn Schmiedebergs Arch Pharmacol (2013) 386:107–24 10.1007/s00210-012-0797-0 23001156 PMC3558669

[154] Kondziella D Brenner E Eyjolfsson EM Markinhuhta KR Carlsson ML Sonnewald U Glial-neuronal interactions are impaired in the schizophrenia model of repeated MK801 exposure. Neuropsychopharmacology (2006) 31:1880–7 10.1038/sj.npp.1300993 16395297

[155] Seeman P Glutamate and dopamine components in schizophrenia. J Psychiatry Neurosci (2009) 34:143–9 19270765 PMC2647567

[156] Field JR Walker AG Conn JP Targeting glutamate synapses in schizophrenia. Trends Mol Med (2011) 17:689–98 10.1016/j.molmed.2011.08.004 21955406 PMC3225651

[157] Matosin N Newell KA Metabotropic glutamate receptor 5 in the pathology and treatment of schizophrenia. Neurosci Biobehav Rev (2012) 37:256–86 10.1016/j.neubiorev.2012.12.005 23253944

[158] Moghadam B Krystal H Capturing the angel in “angel dust”: twenty years of translational neuroscience studies of NMDA receptor antagonists in animals and humans. Schizophr Bull (2012) 38:942–9 10.1093/schbul/sbs075 22899397 PMC3446228

[159] Noetzel MJ Jones CK Conn PJ Emerging approaches for treatment of schizophrenia: modulation of glutamatergic signaling. Discov Med (2012) 14:335–43 23200065 PMC3787874

[160] Bernstein HG Steiner J Bogerts B Glial cells in schizophrenia: pathophysiological significance and possible consequences. Expert Rev Neurother (2009) 9:1059–71 10.1586/ern.09.59 19589054

[161] Steffek AE McCullumsmith RE Haroutunian V Meador-Woodruff JH Cortical expression of glial fibrillary acidic protein and glutamine synthetase is decreased in schizophrenia. Schizophr Res (2008) 103:71–82 10.1016/j.schres.2008.04.032 18562176 PMC3774017

[162] Abi-Dargham A Xu X Thompson JL Gil R Kegeles LS Urban N Increased prefrontal cortical D1 receptors in drug naive patients with schizophrenia: a PET study with [^11^C]NNC112. J Psychopharmacol (2012) 26:794–805 10.1177/0269881111409265 21768159

[163] Arai S Shibata H Sakai M Ninomiya H Iwata N Ozaki N Association analysis of the glutamic acid decarboxylase 2 and the glutamine synthetase genes (GAD2, GLUL) with schizophrenia. Psychiatr Genet (2009) 19:6–13 10.1097/YPG.0b013e328311875d 19125103

[164] Martins-de-Souza D Schmitt A Röder R Lebar M Schneider-Axmann T Falkai P Sex-specific proteome differences in the anterior cingulate cortex of schizophrenia. J Psychiatr Res (2010) 44:989–91 10.1016/j.jpsychires.2010.03.003 20381070

[165] Kalkman HQ Circumstantial evidence for a role of glutamine-synthetase in suicide. Med Hypotheses (2011) 76:905–7 10.1016/j.mehy.2011.03.005 21435795

[166] Bernstein HG Tausch A Wagner R Steiner J Seeleke P Walter M Disruption of glutamate-glutamine-GABA cycle significantly impacts on suicidal behaviour: survey of the literature and own findings on glutamine synthetase. CNS Neurol Disord Drug Targets (2013) 12:900–13 10.2174/18715273113129990091 24040807

[167] Quincozes-Santos A Bobermin LD Tonial RP Bambini-Junior V Riesgo R Gottfried C Effects of atypical (risperidone) and typical (haloperidol) antipsychotic agents on astroglial functions. Eur Arch Psychiatry Clin Neurosci (2010) 260:475–81 10.1007/s00406-009-0095-0 20041330

[168] Sershen H Balla A Aspromonte JM Xie S Cooper TB Javitt DC Characterization of interactions between phencyclidine and amphetamine in rodent prefrontal cortex and striatum: implications in NMDA/glycine-site-mediated dopaminergic dysregulation and dopamine transporter function. Neurochem Int (2008) 52:119–29 10.1016/j.neuint.2007.07.011 17716783

[169] Harte M O’Connor WT Evidence for a selective prefrontal cortical GABA_B_ receptor-mediated inhibition of glutamate release in the ventral tegmental area: a dual probe microdialysis study in the awake rat. Neuroscience (2005) 130:215–22 10.1016/j.neuroscience.2004.08.045 15561437

[170] Díaz-Cabiale Z Vivó M Del Arco A O’Connor WT Harte MK Müller CE Metabotropic glutamate mGlu5 receptor-mediated modulation of the ventral striopallidal GABA pathway in rats. Interactions with adenosine A_2A_ and dopamine D_2_ receptors. Neurosci Lett (2002) 324:154–8 10.1016/S0304-3940(02)00179-9 11988350

[171] Wassef A Baker J Kochan LD GABA and schizophrenia: a review by basic science and clinical studies. J Clin Psychopharmacol (2003) 23:601–40 10.1097/01.jcp.0000095349.32154.a5 14624191

[172] Lisman JE Coyle JT Green RW Javitt DC Benes FM Heckers S Circuit-based framework for understanding neurotransmitter and risk gene interactions in schizophrenia. Trends Neurosci (2008) 31:234–42 10.1016/j.tins.2008.02.005 18395805 PMC2680493

[173] Santana N Mengod G Articas F Quantitative analysis of the expression of dopamine D_1_ and D_2_ receptors in pyramidal and GABAergic neurons of the rat prefrontal cortex. Cereb Cortex (2009) 19:849–60 10.1093/cercor/bhn134 18689859

[174] Ferraro L Tomasini MC Fuxe K Agnati LF Mazza R Tanganelli S Mesolimbic dopamine and cortico-accumbens glutamate afferents as major targets for the regulation of the ventral striato-pallidal GABA pathways by neurotensin peptides. Brain Res Rev (2007) 55:144–54 10.1016/j.brainresrev.2007.03.006 17448541

[175] Yamamura S Ohoyama K Hamaguchi M Nakagawa D Suzuki D Matsumoto E Effects of zotepine on extracellular levels of monoamine, GABA and glutamate in rat prefrontal cortex. Br J Pharmacol (2009) 157:656–65 10.1111/j.1476-5381.2009.00175.x 19371334 PMC2707977

[176] Scarr E Gibbons AS Neo J Udawela M Dean B Cholinergic connectivity: it’s implications for psychiatric disorders. Front Cell Neurosci (2013) 7:55 10.3389/fncel.2013.00055 23653591 PMC3642390

[177] Lester DB Rogers TD Blaha CD Acetylcholine-dopamine interactions in the pathophysiology and treatment of CNS disorders. CNS Neurosci Ther (2010) 16:137–62 10.1111/j.1755-5949.2010.00142.x 20370804 PMC6493877

[178] Buchanan RW Freedman R Javitt DC Abi-Dargham A Lieberman JA Recent advances in the development of novel pharmacological agents for the treatment of cognitive impairments in schizophrenia. Schizophr Bull (2007) 33:1120–30 10.1093/schbul/sbm083 17641146 PMC2632365

[179] Bortz DM Mikkelesen JD Bruno JP Localized infusion of the partial alpha 7 nicotinic receptor agonist SSR180711 evoke rapid and transient increases in prefrontal glutamate release. Neuroscience (2013) 255:55–67 10.1016/j.neuroscience.2013.09.047 24095692

[180] Mackowick KM Barr MS Wing VC Rabin RA Ouellet-Plamondon C George TP Neurocognitive endophenotypes in schizophrenia: modulation by nicotinic receptor systems. Prog Neuro Psychopharmacol Biol Psych (2013). 10.1016/j.pnbp.2013.07.010 23871750 PMC3851927

[181] Seutin V Review dopaminergic neurons: much more than dopamine? Br J Pharmacol (2005) 146:167–9 10.1038/sj.bjp.0706328 16025140 PMC1576274

[182] Meltzer HY Massey BW The role of serotonin receptors in the action of atypical antipsychotic drugs. Curr Opin Pharmacol (2011) 11:59–67 10.1016/j.coph.2011.02.007 21420906

[183] Schreiber R Newman-Tancredi A Improving cognition in schizophrenia with antipsychotics that elicit neurogenesis through 5-HT 1A receptor activation. Neurobiol Learn Mem (2014) 110C:72–80 10.1016/j.nim.2013.12.015 24423786

[184] Hensler JG Artigas F Bortolozzi A Daws LC De Deurawedére P Milan L Catechol/serotonin interactions: systems thinking for brain function and disease. Adv Pharmacol (2013) 68:167–97 10.1016/B978-0-12-411512-5.00009-9 24054145 PMC3902978

[185] Meltzer HY Elkis H Vanover K Weiner DM van Kammen DP Peters P Pimavanserin, a selective serotonin (5-HT)2A-inverse agonist, enhances the efficacy and safety of risperidone, 2 mg/day, but does not enhance efficacy of haloperidol, 2 mg/day: comparison with reference dose risperidone, 6 mg/day. Schizophr Res (2012) 141:144–52 10.1016/j.schres.2012.07.029 22954754

[186] Borroto-Escuela DO Romero-Fernandez W Tarakanov AO Marcellino D Ciruela F Agnati LF Dopamine D2 and 5-hydroxytryptamine 5-HT_2A_ receptors assemble into functionally interacting heterodimers. Biochem Biophys Res Commun (2010) 401:605–10 10.1016/j.bbrc.2010.09.110 20888323

[187] Guo Y Zhang H Chen X Cai W Cheng J Yang Y Evaluation of the antipsychotic effect of bi-acetylated L-stepholidine (l-SPD-A), a novel dopamine and serotonin receptor dual ligand. Schizophr Res (2009) 115:41–9 10.1016/j.schres.2009.08.002 19744833

[188] Klejbor I Kucinski A Wersinger SR Corso T Spodnik JH Dziewiatkowski J Serotonergic hyperinnervation and effective serotonin blockade in an FGF receptor developmental model of psychosis. Schizophr Res (2009) 113:308–21 10.1016/j.schres.2009.06.006 19570652 PMC4681496

[189] Olijslagers JE Werkman TR McCreary AC Kruse CG Wadman WJ Modulation of midbrain dopamine neurotransmission by serotonin, a versatile interaction between neurotransmittters and significance for antipsychotic drug action. Curr Neuropharmacol (2006) 4:59–88 18615139 10.2174/157015906775203020 PMC2430681

[190] Arranz MJ de Leon J Pharmacogenetics and pharmacogenomics of schizophrenia: a review by the last decade of research. Mol Psychiatry (2007) 12:707–47 10.1038/sj.mp.4002009 17549063

[191] Alex KD Pehek EA Pharmacologic mechanisms of serotonergic regulation of dopamine neurotransmission. Pharmacol Ther (2007) 113:296–320 10.1016/j.pharmthera.2006.08.004 17049611 PMC2562467

[192] McCreary AC Glennon JC Ashby CR Jr Meltzer HY Li Z Reinders J-H SLV313 (1-(2,3-dihydro-benzo[1,4]dioxin-5-yl)-4-[5-(4-fluro-phenyl)-pyridin-3-ylmethyl]-piperazine monohydrochloride): a novel dopamine D_2_ receptor antagonist and 5-HT_1A_ receptor agonist potential antipsychotic drug. Neuropsychopharmacology (2007) 32:78–94 10.1038/sj.npp.1301098 16710314

[193] Bhattacharyya S Raote I Bhattacharyya A Miledi R Panicker MM Activation, internalization, and recycling of the serotonin 2A receptor by dopamine. Proc Natl Acad Sci U S A (2006) 103:15248–53 10.1073/pnas.0606578103 17005723 PMC1622808

[194] Meltzer HY Li Z Kaneda Y Ichikawa J Serotonin receptors: their key role in drugs to treat schizophrenia. Prog Neuropsychopharmacol Biol Psychiatry (2003) 27:1159–72 10.1016/j.pnpbp.2003.09.010 14642974

[195] McMahon LR Cunningham KA Role of 5-HT_2A_ and 5-HT_2B/2C_ receptors in the behavioral interactions between serotonin and catecholamine reuptake inhibitors. Neuropsychopharmacology (2010) 24:319–29 10.1016/S0893-133X(00)00206-2 11166521

[196] Gao M Chu H-Y Jin G-Z Zhang Z-J Wu J Zhen X-C L-stepholidine-induced excitation of dopamine neurons in rat ventral tegmental area is associated with its 5-HT1A receptor partial agonist activity. Synapse (2011) 65:379–87 10.1002/syn.20855 20803620

[197] Tarazi FI Neil JC The preclinical profile of asenapine: clinical relevance for the treatment of schizophrenia and bipolar mania. Expert Opin Drug Discov (2013) 8:93–103 10.1517/17460441.2013.738193 23121334

[198] Paz RD Tardito S Atzori M Tseng KY Glutamatergic dysfunction in schizophrenia: from basic neuroscience to clinical psychopharmacology. Eur Neuropsychopharmacol (2008) 18:773–86 10.1016/j.euroneuro.2008.06.005 18650071 PMC2831778

[199] Stone JM Glutamatergic antipsychotic drugs: a new dawn in the treatment of schizophrenia? Ther Adv Psychopharmacol (2011) 1:5–18 10.1177/2045125311400779 23983922 PMC3736896

[200] Leroy C Karila L Martinot JL Lukasiewicz M Duchesnay E Comtat C Striatal and extrastriatal dopamine transporter in cannabis and tobacco addiction: a high-resolution PET study. Addict Biol (2012) 17:981–90 10.1111/j.1369-1600.2011.00356.x 21812871

[201] Coyle JT Basu A Benneyworth M Balu D Konopaske G Glutamatergic synaptic dysregulation in schizophrenia. Handb Exp Pharmacol (2013) 213:267–95 10.1007/978-3-642-25758-2_10 23027419 PMC4817347

[202] Marek GJ Behl B Bespalov AY Gross G Lee Y Schoemaker H Glutamatergic (N-methyl-D-aspartate receptor) hypofrontality in schizophrenia: too little juice or a miswired brain? Mol Pharmacol (2010) 77:317–26 10.1124/mol.109.059865 19933774

[203] Rubio MD Drummond JB Meador-Woodruff J-H Glutamate receptor abnormalities in schizophrenia: implications for innovative therapies. Biomol Ther (Seoul) (2012) 20:1–18 10.4062/biomolther.2012.20.1.001 24116269 PMC3792192

[204] Carlsson A Carlsson ML A dopaminergic deficit hypothesis of schizophrenia: the path to discovery. Dialogues Clin Neurosci (2006) 8:137–42 16640125 10.31887/DCNS.2006.8.1/acarlsson PMC3181749

[205] Carlsson ML Carlsson A Adaptive properties and heterogeneity of dopamine D (2) receptors-pharmacological implications. Brain Res Rev (2008) 58:374–8 10.1016/j.brainresrev.2007.09.007 18511124

[206] Meltzer HY Huang M In vivo actions of atypical antipsychotic drug on serotonergic and dopaminergic systems. Prog Brain Res (2008) 172:177–97 10.1016/S0079-6123(08)00909-6 18772033

[207] Seeman P Battaglia G Corti C Corsi C Bruno V Glutamate receptor mGlu2 and mGlu3 knockout sriata are dopamine supersensitive, with elevated D2 (High) receptors and marked supersensitivity to the dopamine agonist (+)PHNO. Synapse (2009) 63:247–51 10.1002/syn.20607 19084908

[208] Carlsson ML Burstein ES Kloberg A Hansson S Schedwin A Nilsson M I. In vivo evidence for partial agonist effects of (-)-OSU6162 and (+)-OSU6162 on 5-HT2A serotonin receptors. J Neural Transm (2011) 118(11):1511–22 10.1007/s00702-011-0704-8 21874578

[209] Meltzer HY Serotonergic mechanisms as targets for existing and novel antipsychotics. Handb Exp Pharmacol (2012) 212:87–124 10.1007/978-3-642-25761-2_4 23129329

[210] Purkayastha S Ford J Kanjilal B Diallo S Del Rosario Inigo J Neuwirth L Clozapine functions through the prefrontal cortex serotonin 1A receptor to heighten neuronal activity via calmodulin kinase II-NMDA-receptor interactions. J Neurochem (2012) 120:396–407 10.1111/j.1471-4159.2011.07565.x 22044428 PMC3253201

[211] Carlsson ML Martin P Nilsson M Waters S Waters N The 5-HT2A receptor antagonist M10097 is more effective in counteracting NMDA antagonist-than dopamine agonist-induced hyperactivity in mice. J Neural Transm (1999) 106:123–9 10.1007/s007020050144 10226932

[212] Beaulieu J-M Del’Guidice T Sotnikova TD Lemasson M Gainetdinov RR Beyond cAMP: the regulation of Akt and GSK3 by dopamine receptors. Front Mol Neurosci (2011) 4:38 10.3389/fnmol.2011.00038 22065948 PMC3206544

[213] Souza BR Romano-Silva MA Tropepe V Dopamine D_2_ receptor activity modulates Akt signaling and alters GABAergic neuron development and motor behavior in zebrafish larvae. J Neurosci (2011) 31:5512–25 10.1523/JNEUROSCI.5548-10.2011 21471388 PMC6622698

[214] Benes FM The role of stress and dopamine-GABA interactions in the vulnerability for schizophrenia. J Psychiatr Res (1997) 1997:257–75 10.1016/S0022-3956(96)00044-1 9278189

[215] Arnsten AFT Prefrontal cortical network connections: key site of vulnerability in stress and schizophrenia. Int J Dev Neurosci (2011) 29:215–23 10.1016/j.ijdevneu.2011.02.006 21345366 PMC3115784

[216] van der Stelt M Di Marzo V The endocannabinoid system in the basal ganglia and in the mesolimbic reward system: implications for neurological and psychiatric disorders. Eur J Pharmacol (2003) 480:133–50 10.1016/j.ejphar.2003.08.101 14623357

[217] Fernandez-Ruiz J Hernandez M Ramos JA Cannabinoid-dopamine interaction in the pathophysiology and treatment of CNS disorders. CNS Neurosci Ther (2010) 16:e72–91 10.1111/j.1755-5949.2010.00144.x 20406253 PMC6493786

[218] Behan AT Hryniewiecka M O’Tuathaigh CM Kinsella A Cannon M Karayiorgou M Chronic adolescent exposure to delta-9-tetrahydrocannabinoid and GABAergic pathways. Neuropsychopharmacology (2012) 37:1773–83 10.1038/npp.2012.24 22434221 PMC3358747

[219] Hains AB Arnsten FT Molecular mechanisms of stress-induced prefrontal cortical impairment: implications for mental illness. Learn Mem (2008) 15:551–64 10.1101/lm.921708 18685145

[220] Lipina TV Niwa M Jaaro-Peled H Fletcher PJ Seeman P Sawa A Enhanced dopamine function in DISC1-L100P mutant mice: implications for schizophrenia. Genes Brain Behav (2010) 9:777–89 10.1111/j.1601-183X.2010.00615.x 20618446

[221] Broadhurst CL Crawford MA Cunnane SC Parkington JE Schmidt WF Brain-specific lipids from marine, lacustrine, or terrestrial food resources: potential impact on early African *Homo sapiens*. Comp Biochem Physiol B Biochem Mol Biol (2002) 131:653–73 10.1016/S1096-4959(02)00002-7 11923081

[222] Marean CW Bar-Matthews M Bernatchez J Fisher E Goldberg P Herries AI Early human use of marine resources and pigment in South Africa during the Middle Pleistocene. Nature (2007) 499:905–8 10.1038/nature06204 17943129

[223] Previc F The Dopaminergic Mind in Human Evolution and History. New York, NY: Cambridge University Press (2009).

[224] Previc FH Thyroid hormone production in chimpanzees and humans: implications for the origins of human intelligence. Am J Phys Anthropol (2002) 118:402–3 10.1002/ajpa.10095 12124921

[225] Mattson MP Evolutionary aspects of human exercise – born to run purposefully. Ageing Res Rev (2012) 11:347–52 10.1016/j.arr.2012.01.007 22394472 PMC3356485

