# Corrigendum: The Role of Dopamine in Schizophrenia from a Neurobiological and Evolutionary Perspective: Old Fashioned, but Still in Vogue

**DOI:** 10.3389/fpsyt.2014.00110

**Published:** 2014-08-27

**Authors:** Ralf Brisch, Arthur Saniotis, Rainer Wolf, Hendrik Bielau, Hans-Gert Bernstein, Johann Steiner, Bernhard Bogerts, Katharina Braun, Zbigniew Jankowski, Jaliya Kumaratilake, Maciej Henneberg, Tomasz Gos

**Affiliations:** ^1^Department of Forensic Medicine, Medical University of Gdańsk, Gdańsk, Poland; ^2^School of Medical Sciences, The University of Adelaide, Adelaide, SA, Australia; ^3^Centre for Evolutionary Medicine, University of Zurich, Zurich, Switzerland; ^4^Department of Psychiatry and Psychotherapy, Ruhr University Bochum, Bochum, Germany; ^5^Department of Psychiatry, Otto-von-Guericke-University of Magdeburg, Magdeburg, Germany; ^6^Department of Zoology/Developmental Neurobiology, Institute of Biology, Otto-von-Guericke-University of Magdeburg, Magdeburg, Germany; ^7^Biological Anthropology and Comparative Anatomy Research Unit, School of Medical Sciences, The University of Adelaide, Adelaide, SA, Australia

**Keywords:** dopamine, schizophrenia, cognition, glutamate, dopamine receptors, cannabis, animal models of schizophrenia, evolution of the human brain

The author Jaliya Kumaratilake’s name was misspelled and the author Katharina Braun’s name was wrongly displayed in the review “The role of dopamine in schizophrenia from a neurobiological and evolutionary perspective: old fashioned, but still in vogue”. The correct citation for the review should be as follows:

Brisch R, Saniotis A, Wolf R, Bielau H, Bernstein H-G, Steiner J, Bogerts B, Braun K, Jankowski Z, Kumaratilake J, Henneberg M, Gos T (2014) The role of dopamine in schizophrenia from a neurobiological and evolutionary perspective: old fashioned, but still in vogue. Front Psychiatry **5**:47. doi: 10.3389/fpsyt.2014.00047.

The original article has been updated.

## Conflict of Interest Statement

The authors declare that the research was conducted in the absence of any commercial or financial relationships that could be construed as a potential conflict of interest.

